# Hepatitis B virus infection in undocumented immigrants and refugees in Southern Italy: demographic, virological, and clinical features

**DOI:** 10.1186/s40249-016-0228-4

**Published:** 2017-02-09

**Authors:** Nicola Coppola, Loredana Alessio, Luciano Gualdieri, Mariantonietta Pisaturo, Caterina Sagnelli, Carmine Minichini, Giovanni Di Caprio, Mario Starace, Lorenzo Onorato, Giuseppe Signoriello, Margherita Macera, Italo Francesco Angelillo, Giuseppe Pasquale, Evangelista Sagnelli

**Affiliations:** 1 Department of Mental Health and Public Medicine, Section of Infectious Diseases, Second University of Naples, Via L. Armanni 5, 80133 Naples, Italy; 2 Medical Center, Social center “ex Canapificio”, Caserta, Italy; 3 Medical Center, Center for the wardship of the immigrants, Naples, Italy; 4 Medical Center, Welcome center ‘La tenda di Abramo’, Caserta, Italy; 5 Infectious Diseases Unit, AORN Sant’Anna e San Sebastiano, Caserta, Italy; 6 Department of Clinical and Experimental Medicine and Surgery, Second University of Naples, Naples, Italy; 7 Medical center, Center of missionary nuns of carithy, Naples, Italy; 8 Department of Mental Health and Public Medicine, Section of Statistic, Second University of Naples, Naples, Italy; 9 Department of Experimental Medicine, Second University of Naples, Naples, Italy

**Keywords:** Hepatitis B, Chronic hepatitis B virus infection, Immigration, Illegal immigrants, Refugees, Italy

## Abstract

**Background:**

The data on hepatitis b virus (HBV) infection in immigrants population are scanty. The porpoise of this study was to define the demographic, virological, and clinical characteristics of subjects infected with HBV chronic infection in a cohort of immigrants living in Naples, Italy.

**Methods:**

A screening for HBV infection was offered to 1,331 immigrants, of whom 1,212 (91%) (831 undocumented immigrants and 381 refugees) accepted and were screened for hepatitis B surface antigen (HBsAg) and anti-hepatitis B core antibody (HBc). Those found to be HBsAg positive were further investigated at third-level infectious disease units.

**Results:**

Of the 1,212 immigrants screened, 116 (9.6%) were HBsAg positive, 490 (40.4%) were HBsAg negative/anti-HBc positive, and 606 (50%) were seronegative for both. Moreover, 21 (1.7%) were anti-human immunodeficiency virus positive and 45 (3.7%) were anti-hepatitis C virus positive. The logistic regression analysis showed that male sex (*OR*: 1.79; 95%*CI*: 1.28–2.51), Sub-Saharan African origin (*OR*: 6.18; 95%*CI*: 3.37–11.36), low level of schooling (*OR*: 0.96; 95%*CI*: 0.94–0.99), and minor parenteral risks for acquiring HBV infection (acupuncture, tattoo, piercing, or tribal practices, *OR*: 1.54; 95%*CI*: 1.1–2.16) were independently associated with ongoing or past HBV infection. Of the 116 HBsAg-positive immigrants, 90 (77.6%) completed their diagnostic itinerary at a third-level infectious disease unit: 29 (32.2%) were asymptomatic non-viremic HBsAg carriers, 43 (47.8%) were asymptomatic viremic carriers, 14 (15.6%) had chronic hepatitis, and four (4.4%) had liver cirrhosis, with superimposed hepatocellular carcinoma in two.

**Conclusions:**

The data illustrate the demographic, clinical and virological characteristics of HBV infection in immigrants in Italy and indicate the need for Italian healthcare authorities to enhance their support for providing screening, HBV vaccination, treatment, and educational programs for this populations.

**Electronic supplementary material:**

The online version of this article (doi:10.1186/s40249-016-0228-4) contains supplementary material, which is available to authorized users.

## Multilingual abstracts

Please see Additional file 1 for translation of the abstract into the five official working languages of the United Nations.

## Background

The hepatitis B virus (HBV) is the most common agent of hepatitis worldwide, with around 350–400 million people chronically infected [1] and 600,000 deaths reported each year due to a fulminant course of acute hepatitis B (AHB) or, more frequently, to liver decompensation in hepatitis B surface antigen (HBsAg)-positive patients with cirrhosis or hepatocellular carcinoma (HCC) [2–4]. The HBV is transmitted from infected mothers to their new-born babies at birth and in childhood, and in adulthood by parenteral (unsafe blood transfusion, intravenous drug use, surgery, dialysis, tattooing, piercing) or sexual (heterosexual or homosexual) routes. The level of HBV endemicity differs from one country to another: it is low in Western Europe, USA, Canada, and some South American and Northern African countries (with an HBsAg chronic carrier rate below 2%); intermediate in Eastern Europe, Central Asia, and some Eastern Asian countries (from 2 to 8%); and high in some Asian and Sub-Saharan African countries and in Alaska (above 8%) [1]. In Italy, HBsAg seroprevalence is estimated to be around 1% and the yearly incidence rate of AHB is nearly 1/100,000 inhabitants [3, 4].

Due to the socioeconomic and political crises in Northern Africa, Sub-Saharan Africa (SSA), Eastern Europe, and Central and Eastern Asia in recent decades, Western countries have become lands of immigration from these subcontinents with intermediate or high HBV, hepatitis C virus (HCV), and human immunodeficiency virus (HIV) endemicities. At present, approximately 5.4 million legal immigrants live in Italy, making up 8.2% of the resident population (http://www.dossierimmigrazione.it/docnews/file/Scheda%20Dossier%202015(4).pdf). In addition, Italian immigration authorities estimate that around 500,000 undocumented immigrants live in Italy at present, prevalently coming from Northern Africa and SSA, Eastern Europe, and Central and Eastern Asia [5, 6]. The immigrant population is prevalently young, sexually active [7, 8], and has broken family ties. They often have no fixed abode or live in crowded homes; are not socially integrated due to language, cultural, and socioeconomic barriers [9]; and consequently, have limited access to healthcare services.

In our previous study, conducted from January 2012 to June 2013, we screened 882 immigrants; the resulting HBsAg seroprevalence was 8% [10]. In the present study, we report on the demographic, virological, and clinical characteristics of 116 HBsAg-positive subjects, after screening 1,212 undocumented immigrants and low-income refugees from January 2012 to December 2014 using the same methodology as in the previous study [10].

## Methods

### Patients

#### Study design

The design of this study was extensively described in a previous paper [10]. Briefly, this is a multicentre prospective study with the participation of six centres: three in Naples (two first-level clinical centres and one tertiary unit of infectious diseases) and three in Caserta (two first-level clinical centres and one tertiary unit of infectious diseases). All immigrants—undocumented immigrants and low-income refugees—consecutively seen for a clinical consultation at one of the four first-level centres from January 2012 to December 2014 were enrolled in the study. Undocumented immigrant and low-income refugee populations living in Italy have similar characteristics: they are all prevalently young; not integrated due to language, cultural, and social barriers; and have low incomes, most frequently from casual work.

#### Study sites

The first-level clinical centres are hospital centres of the national healthcare system or clinical centres of international charity organizations supported by the national healthcare system, with proven experience in clinical, psychological, and legal management of vulnerable groups, such as undocumented immigrants, low-income refugees, the homeless, and alcoholics. Each first-level clinical centre is an outpatients clinic providing general medical services. The most frequent pathological conditions inducing undocumented immigrants and refugees to refer to one of these centres are lumbago, headaches, pruritus, coughs, high blood pressure, and allergy symptoms.

#### Screening of patients

During a clinical consultation, a physician from the clinical centre and a cultural mediator explained to the immigrants the importance of testing for HBV, HCV, and HIV serum markers, and offered them to be screened free of charge, in anonymity (centre number, patient number), and in full accordance with the privacy law. Acceptance of screening and a signed informed consent, written in the immigrant’s native language, was obtained on a voluntary basis from more than 91% (1,212) of the 1,331 undocumented immigrants and low-income refugees at one of the four first-level clinical units during the study period. These were the subjects who participated in the study.

#### Questionnaire

An anonymous questionnaire collecting information on the demographics (age, sex, race/ethnicity, place of birth, language); socioeconomic status (education, annual household income); environmental factors (alcohol, diet, etc); and clinical data and risk factors for acquiring HBV, HCV (sexual contact, drug, use, surgery, etc), and HIV infections was completed by the 1212 subjects who agreed to participate in this study.

#### Serum sampling and clinical definitions

For all subjects enrolled, a serum sample was obtained to test for HBsAg, total anti- hepatitis B core antibody (HBc), anti-HCV, anti-HIV, and serum aminotransferases. HBsAg positivity was considered a marker of ongoing HBV infection, and HBsAg negativity/anti-HBc positivity as markers of a past HBV infection; HBsAg/anti-HBc-negative subjects were considered as having no HBV infection.

The HBsAg-positive subjects were referred for further investigation, monitoring, and possible treatment to one of the two tertiary units of infectious diseases, both of which are affiliated with the Second University of Naples and have cooperated for nearly 15 years in several clinical investigations on HBV infection using the same clinical approach and laboratory methods [11, 12]. Each HBsAg-positive subject was assigned to the care of a cultural mediator, who, acting as a support, assisted him/her at the third-level clinical centre throughout the monitoring and/or treatment period.

HBsAg-positive patients were classified as asymptomatic carriers when, in the absence of clinical, biochemical, and ultrasound signs of chronic liver disease, alanine aminotransferase (ALT) values were persistently normal. Chronic hepatitis was diagnosed based on liver histology or, if not performed, based on abnormal ALT values. Liver cirrhosis was diagnosed with a liver biopsy or, if not performed, from the presence of unequivocal clinical, biochemical, and ultrasound signs [13]. The diagnosis of HCC was based on histology, imaging techniques, or biochemical parameters (α1-feto protein greater than 400 ng/mL) [14].

### Methods

Serum samples were tested for HBsAg, anti-HCV, anti-HIV, total anti-HBc, and anti-hepatitis B surface antibody (HBs) using commercial immunoenzymatic assays (Abbott Laboratories, North Chicago, IL, USA: AxSYM® HBsAg (v2) M/S for HBsAg, AxSYM® HCV (v3) for anti-HCV, AxSYM® HIV 1/2 Combo for HIV, AxSYM® CORE™ (v2) for total anti-HBc, and AxSYM® AUSAB® for anti-HBs). Anti-HIV reactivity was always confirmed by a western blot assay (Genelabs Diagnostics, Science Park Drive, Singapore), which identifies both HIV-1 and HIV-2 strains.

Serum HBV-DNA levels were determined by real-time polymerase chain reaction (PCR) with a detection limit of 20 copies/mL, as previously described [15]. The HBV genotype was determined in HBV DNA positive samples, as previously described [16].

### Statistical analysis

Continuous variables were summarized as mean and standard deviations (SD), and categorical variables as absolute and relative frequencies. Differences in mean values were evaluated using the Student’s t-test, while the chi-square test was used for categorical variables. The odds ratio (*OR*), with a 95% confidence interval (*CI*), was estimated using a logistic regression model to identify possible independent associations between the presence of HBV infection (ongoing or past) with sex, age, country of origin, years of schooling, and possible risk factors for its acquisition. A *P* < 0.05 was considered to be statistically significant.

### Ethics approval

The Ethics Committee of the Azienda Ospedaliera Universitaria of the Second University of Naples (214/2012) approved this study. Signed informed consent, written in the immigrant’s native language, was obtained on a voluntary basis from more than 91% (1,212) of the 1,331 undocumented immigrants and low-income refugees at one of the four first-level clinical units during the study period. All patients signed an informed consent for the collection and storage of biological samples and for the anonymous use of their data for research purposes these subjects participated in the study.

## Results

The initial demographic and serological data pertaining to the 1,212 immigrants investigated in this study are shown in Table 1. The subjects were mostly young (median age 32 years, range 12–74 years), prevalently males (75.2%), and had been living in Italy for a mean period of 50.3 months (SD ± 53.0). Of the 1,212 immigrants, 668 (55.1%) came from SSA, 237 (19.5%) from Eastern Europe, 88 (7.3%) from Northern Africa, 207 (17.1%) from Asia, 10 (0.8%) from South America, and 2 (0. 2%) did not state their country of origin (see Table 1).

**Table 1:** Demographic and initial characteristics of the 1,212 immigrants enrolled in the study

	Total
N° of patients	1,212
Age, years, median (range)	32 (12–74)
Males, n° (%)	911 (75.2)
Legal status, n° (%):	
Undocumented immigrants	831 (68.6)
Low-income refugees	381 (31.4)
In Italy for months, mean + SD	50.3 ± 53.0
Place of origin, n° (%)	
Eastern Europe	237 (19.5)
Africa	756 (62.4)
Asia	207 (17.1)
America	10 (0.8)
Not stated	2 (0.2)
HBV serological status, n° (%)	
HBsAg positive, total number	116 (9.6)
HBsAg positive	113 (9.3)
HBsAg positive/anti-HIV positive	2 (0.2)
HBsAg/anti-HCV/anti-HIV positive	1 (0.1)
HBsAg negative/anti-HBc positive	490 (40.4)
HBsAg/anti-HBc negative	606 (50.0)

Of the 1 212 immigrants, 116 (9.6%) were HBsAg positive (113 with HBsAg alone, two had HBsAg and were anti-HIV positive, and one was HBsAg, anti-HCV, and anti-HIV positive); 490 (40.4%) were HBsAg negative/anti-HBc positive, and 606 (50%) were HBsAg/anti-HBc negative (see Table 1). Of the 1 096 HBsAg-negative subjects, 40 (3.6%) were anti-HCV positive, 14 (1.3%) were anti-HIV positive, and 4 (0.4%) were anti-HCV/anti-HIV positive. Thus, 21 (1.7%) subjects were anti-HIV positive and 45 (3.7%) were anti-HCV positive. All subjects were unaware of their serological status.

The demographic and initial characteristics of the 1 212 subjects were also analysed according to their HBV serological condition. Compared with the HBsAg/anti-HBc-negative subjects, HBsAg-positive or HBsAg-negative/anti-HBc-positive patients were more frequently males (81.5 and 80.8% vs. 70%, *P* = 0.001) and more frequently came from SSA (76.5 and 70.4% vs. 37.4%, *P* = 0.001). The HBsAg-positive subjects had fewer years of schooling than the HBsAg/anti-HBc-negative (4.5 ± 3.9 vs. 8.1 ± 5.3, *P* = 0.000) and the HBsAg-negative/anti-HBc-positive (12.9 ± 2.9 years, *P* = 0.000) patients (see Table 2).

**Table 2:** Demographic and initial characteristics of the 1,212 immigrants enrolled in the study, according to HBV serology

	HBsAg positive	HBsAg negative/anti-HBc positive	HBsAg/anti-HBc negative	HBsAg positive + HBsAg negative/anti-HBc positive vs. negative for both
N° of patients	116	490	606	
Age, years, mean ± SD	32.4 ± 8 α	34 ± 10 b	33.7 ± 11 c	0.50
Males, n° (%)	97 (81.5)	396 (80.8)	420 (70)	0.000
Legal status				0.9
Undocumented immigrants	78 (71.6)	339 (69.2)	414 (68)	
Low-income refugees	38 (28.4)	151 (30.8)	192 (31.7)	
Country of origin, n° (% by row)				
Eastern Europe, 233 cases	14 (6.0)	73 (31.3)	146 (62.7)	0.000
Northern Africa, 87 cases	3 (3.5)	17 (19.5)	67 (77.0)	
SSA, 665 cases	93 (14.0)	345 (51.9)	227 (34.1)	
India-Pakistan area, 175 cases	5 (2.9)	46 (26.3)	124 (70.8)	
Others, 52 cases	1 (1.9)	9 (17.3)	42 (80.8)	
In Italy for months, mean ± SD	42.2 ± 50	52.7 ± 51	51 ± 55.5	0.92
Years of schooling, mean ± SD	4.5 ± 3.9	12.9 ± 2.9	8.1 ± 5.3	0.000
n° (%) with alcohol intake	22 (16)	102 (20.8)	131 (21.6)	0.67
Declared risk factors, n° (% by column)				
Drug addiction	0	5 (1)	3 (0.5)	
Unsafe sexual intercourse	21 (18)	85 (17.3)	113 (18.6)	0.68
Surgery, dental care, abortion	56 (48.3)	247 (50.4)	315 (52)	
Blood transfusion	2 (1.7)	10 (2)	4 (0.7)	
Other parenteral exposure^a^	93 (80)	351 (71.6)	441 (72.8)	
Did not declare risk factors	12 (10.3)	71 (14.5)	92 (15.2)	

^a^Unsafe injection therapy, acupuncture, tattoo, piercing, tribal practices

To identify the factors independently associated with the acquisition of an ongoing or previous HBV infection, a logistic regression analysis was performed with sex, age, country of origin, years of schooling, and sexual and parenteral risk factors as covariates. The analysis identified the male sex (*OR*: 1.79; 95%CI: 1.28–2.51, *P* = 0.001), fewer years of schooling (*OR*: 0.96; 95%*CI*: 0.94–0.99, *P* = 0.007), and a history of acupuncture, tattooing, piercing, or other tribal practices (*OR*: 1.54; 95%*CI*: 1.1–2.16, *P* = 0.011) as being independently associated with acquiring a HBV infection. In addition, compared with immigrants from Northern Africa, those from SSA (*OR*: 6.18; 95%*CI*: 3.37–11.36, *P* = 0.000), Asia (*OR*: 2.65; 95%*CI*: 1.35–5.21, *P* = 0.005), and Eastern Europe (*OR*: 2.00; 95%*CI*: 1.02–3.91, *P* = 0.043) more frequently had HBV infection (see Table 3).

**Table 3:** Logistic regression analysis for independent predictors of contact with HBV (HBsAg-positive or HBsAg-negative/anti-HBc-positive status vs. HBsAg/anti-HBc-negative status)

Parameter	*OR*	95%*CI*		*P*
		Lower	Upper	
Gender				
Male vs. female	1.79	1.28	2.51	0.001
Age	1.02	1.01	1.04	0.001
Country of origin				
SSA vs. North Africa	6.18	3.37	11.36	0.000
Eastern Europe vs. North Africa	2.00	1.02	3.91	0.043
Asia vs. North Africa	2.65	1.35	5.21	0.005
America vs. North Africa	0.96	0.07	6.07	0.72
Years of schooling	0.96	0.94	0.99	0.007
Sexual risk factors				
Sexual vs. parenteral exposure^a^	0.73	0.49	1.1	0.13
Risk factors (minor^b^)				
Minor risks vs. other risks	1.54	1.1	2.16	0.011

^a^Drug addiction, surgery, dental care, abortion, blood transfusion

^b^Acupuncture, tattoo, piercing, tribal practices

**Fig. 1:**
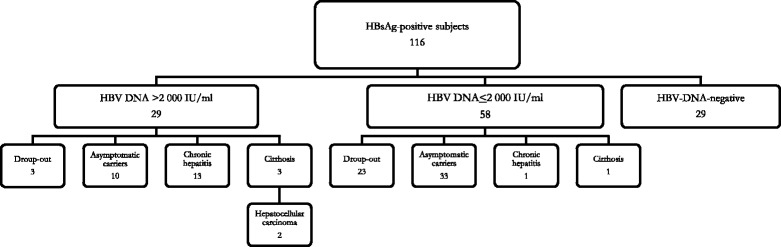
Clinical diagnosis of the 116 HBsAg-positive subjects

All HBsAg-positive subjects were referred to one of the two tertiary units of infectious diseases for further investigation, monitoring, and possible treatment. Of the 116 HBsAg-positive subjects, 29 (25%) were serum HBV DNA negative with normal aminotransferase serum values in two determinations at a 3–6 month interval and were considered asymptomatic non-viremic HBsAg carriers. Hepatitis B virus DNA was detected in 87 (75%) HBsAg-positive subjects, with a HBV DNA load ≤ 2 000 IU/ml in 58 (50%) and >2 000 IU/ml in the remaining 29 (25%). However, three (10.3%) of the 29 subjects with a serum HBV load >2 000 IU/ml and 23 (39.7%) of the 58 with a HBV DNA load ≤ 2 000 IU/ml did not complete the diagnostic itinerary (see Fig. 1).

Of the 26 HBsAg-positive subjects with a HBV DNA load >2 000 IU/ml who completed the diagnostic procedures, 10 (38.5%) were considered asymptomatic viremic HBsAg carriers because they showed persistently normal aminotransferase serum values and a normal liver at ultrasound examination; all were anti-hepatitis Be antibody (HBe) positive and had a HBV load between 2 001 and 10 000 IU/ml. Another 13 (50%) showed clinical, laboratory, and US patterns characteristic of chronic hepatitis, and the remaining three (11.5%) had liver cirrhosis, with superimposed HCC in two patients (see Fig. 1).

Of the 35 subjects with serum HBV DNA ≤2 000 IU/ml who completed the diagnostic procedures, 33 (94.3%) were conclusively diagnosed as asymptomatic HBsAg carriers with low viremia, one (2.9%) with chronic hepatitis, and one (2.9%) with liver cirrhosis (see Fig. 1).

Overall, a conclusive diagnosis was obtained for 90 (77.6%) of the 116 HBsAg-positive subjects. Of these, 29 (32.2%) were asymptomatic non-viremic HBsAg carriers, 43 (47.8%) were asymptomatic viremic HBsAg carriers, 14 (15.6%) had chronic hepatitis, and four (4.4%) had liver cirrhosis, with superimposed HCC in two patients. Of these 90 HBsAg-positive subjects, two (2.2%) were anti-Delta positive, six (6.7%) were hepatitis B e antigen (HBeAg) positive, and 84 (93.3%) were anti-HBe positive. The HBV genotype was identified in 47 of the 61 HBV-DNA-positive subjects with a conclusive diagnosis; a low HBV DNA serum concentration did not allow sequencing in 14 cases. Of the 47 genotyped patients, 11 (23.4%) had HBV genotype A, seven (14.9%) had genotype D, 28 (59.6%) had genotype E, and only one (2.1%) had genotype C.

The demographic, serological, and virological characteristics of the 90 HBsAg-positive subjects with a conclusive diagnosis are shown in Table 4, according to the disease stage. Compared with patients with a less active liver disease, those with chronic hepatitis or liver cirrhosis showed a higher viral load and higher aminotransferase serum levels and were more frequently HBeAg positive (see Table 4).

**Table 4:** Demographic, serological, and virological characteristics according to the clinical classifications of the 90 HBsAg-positive subjects with a conclusive clinical diagnosis

	HBsAg pos. non-viremic asymptomatic carriers	HBsAg pos. viremic asymptomatic carriers	HBsAg pos. patients with chronic hepatitis	HBsAg-pos. patients with cirrhosis
N° of patients	29	43	14	4
Age, years, median (range)	32 (22–58)	32.5 (20–55)	32.5 (18–44)	32.5 (25–35)
Males, n° (%)	25 (86)	35 (83.3)	14	4
Status in country, n° (% by row)				
Undocumented immigrants, 63 cases	14 (22.2)	32 (50.8)	13 (20.6)	4 (6.4)
Low-income refugees, 27 cases	15 (52)	11 (26.2)	1 (7)	0
In Italy for month, mean ± SD	35.7 ± 32.7	42 ± 58.6	49.4 ± 28.8	50 ± 52.3
Years of schooling, mean ± SD	3.5 ± 4.2	2.5 ± 4	11.9 ± 19.8	2.7 ± 3.8
Country of origin, n° (% by row)				
Eastern Europe, 10 cases	1 (10.0)	5 (50.0)	4 (40.0)	0
North Africa, 1 case	0	1 (100)	0	0
SSA, 75 cases	27 (36)	35 (46.7)	10 (13.3)	3 (4)
Asia, 4 cases	1 (25)	2 (50)	0	1 (25)
America, no cases	0	0	0	0
Anti-delta-positive, n°(% by column)	0	1 (2.4)	1 (7)	0
HBeAg positive/anti-HBe negative, n° (% by column)	0	0	4 (28.6)	2 (50)
HBeAg negative/anti-HBe positive, n° (% by column)	29 (100)	43 (100)	10 (71.4)	2 (50)
HBV DNA, IU/ml, median (range)	--	620 (12–73 000)	8,500 (2.4E3–1.0E9)	1.2E7 (1.3E–1.7E8)
AST, IU/ml, mean ± SD	18 ± 3.7	23.6 ± 7.2	72.7 ± 82.5	41 ± 22
ALT, IU/ml, mean ± SD	19 ± 4.3	24.7 ± 7	98.5 ± 117.8	53 ± 32.2
HBV genotype, n° (% by row)				
A	//	8 (72.7)	2 (18.2)	1 (9.1)
C		0	0	1 (25)
D		4 (57.1)	3 (42.9)	0
E		18 (64.3)	8 (28.6)	2 (7.1)
Not determined		13 (92.9)	1 (7.1)	0

*AST* aspartate-aminotransferases, *ALT* alanine-aminotransferase

The HBsAg-positive subjects admitted to the present study received treatment or remained untreated in accordance with the current international guidelines [13]. In particular, five of the 14 patients with chronic hepatitis were treated with peginterferon α-2a (180ug once a week) for 12–24 months; a favourable response was observed only in one, a HBV-genotype-A Romanian patient. Another six patients with chronic hepatitis and three of the four cirrhotic patients were treated with nucleos(t)ide analogues: entecavir was given to five cases and tenofovir to four. All nine nucleos(t)ide-analogue-treated patients became serum HBV DNA negative within the 48^th^ week of treatment and remained so after. For the remaining three patients with chronic hepatitis, antiviral treatment was not indicated and they were left untreated. Also untreated was a patient from SSA with advanced liver cirrhosis and multifocal HCC who died after a few weeks of observation.

## Discussion

Despite their long-term stay in Italy, the undocumented immigrants and low-income refugees investigated in this study were poorly integrated due to language, cultural, and socioeconomic barriers. This immigrant population came to Italy from various countries with intermediate or high HBV endemicities and with different socioeconomic, religious, and cultural backgrounds, all of which makes their access to Italian healthcare services difficult. Nevertheless, the presence of skilled physicians and cultural mediators operating in the four first-level centres overcame any language and cultural barriers and allowed successful screening with an over-90% acceptance rate. The rate of the interviewed immigrants who agreed to be screened seems a useful parameter for evaluating the efficacy of screening and representative of the immigrant population. However, a possible bias on the enrolment may not be excluded.

The HBsAg-positive subjects were referred to a tertiary clinical centre to complete their diagnostic itinerary and receive treatment, if indicated. Overall, the strategies used in this study could be recommended for screening undocumented immigrants and low-income refugees in several clinical settings.

In agreement with the recommendations of the Centers for Disease Control and Prevention in Atlanta, USA, the data from our study underscore the need for universal screening for HBV infection for people from countries with an HBsAg prevalence higher than 2% [17]. In fact, the individuals from SSA, who accounted for over half of the subjects in this study, showed an ongoing HBV infection in 11.3% and a past HBV infection in more than half of the cases. The rate of HBsAg positivity observed in this subcontinent is very high, thus suggesting that in most cases HBV infection was acquired early in life, at birth from HBsAg-positive mothers, or in early youth from infected parents or siblings [3, 18, 19]. In addition, the immigrants from Eastern Europe, the India-Pakistan subcontinent, and Northern Africa investigated in this study showed intermediate HBsAg-positivity rates. The prevalences observed in undocumented immigrants and low-income refugees in this study indicate the widespread HBV infection in their countries of origin, since the rate of HBsAg positivity in Italy is estimated to be below 1% [20–23].

Subjects participating in the present study were relatively young, prevalently males, and had been living in Italy for a mean period of four and a half years. All immigrants with an ongoing or previous HBV infection were unaware of their serological status and, compared with the HBsAg/anti-HBc-negative patients, were more frequently males and more frequently from SSA. Accordingly, a logistic regression analysis identified the male sex and Sub-Saharan African origin as independent predictors of a persisting or past HBV infection. The other independent predictors identified in this study were a low level of schooling and the presence of ‘minor’ parenteral risk factors (acupuncture, tattooing, piercing, or tribal practices). Worthy of note is the observation that in our immigrant population, in which the ‘main’ routes of parenteral transmission played a minor role in transmitting HBV infection, the so-called ‘minor’ risk factors were instead identified as being independently associated with transmission. Furthermore, that education plays a major role in the prevention of infectious diseases is once again demonstrated in the present study, as a low level of schooling was independently associated with HBV transmission [4, 24–28].

Referred to one of the two tertiary units of infectious diseases for further investigation, monitoring, and possible treatment, approximately 10% of the subjects with a serum HBV load >2 000 IU/ml and nearly 40% of those with a lower HBV replication did not complete the diagnostic course. This partial success suggests that an improvement in the skills of some cultural mediators is necessary.

A conclusive diagnosis was obtained for 90 of the 116 HBsAg-positive immigrants: 29 were asymptomatic non-viremic HBsAg carriers, 43 were asymptomatic viremic carriers, and 18 had viremic chronic hepatitis or cirrhosis. Moreover, those with a HBV load above 2,000 IU/ml had chronic hepatitis or liver cirrhosis more frequently than those with lower viremia and, conversely, were less frequently asymptomatic viremic HBsAg carriers. These data indicate that, when applying only the HBV DNA serum value of 2,000 IU/ml to distinguish low from high viremic subjects for clinical and therapeutic decisions, as suggested by the current international guidelines [13], over 5% of the low viremic and nearly 40% of the high viremic immigrants in the present study could have been misclassified. It is very likely that the current international guidelines do not consider patients with HBV-genotype E chronic hepatitis, a genotype detected in recent years mostly in populations from SSA and which predominated in this study. We believe that more attention should be given to this genotype, the epidemiological impact of which is steadily increasing [29–33].

Literature on the treatment of HBV-related chronic hepatitis in immigrants is scanty [34–36] and does not allow for any conclusive evaluation. All chronic hepatitis and cirrhosis patients in the present study were considered for anti-HBV treatment and, in accordance with the current national guidelines [13, 37, 38], some were left untreated and some were treated with either peginterferon α-2a or nucleos(t)ide analogues, with results similar to those observed for the local Italian population [39–42].

## Conclusions

The present investigation provides interesting information on the presence of HBV infection in undocumented immigrants and refugee populations from different geographical areas [43–47], and could be useful for devising healthcare strategies in Italy. Virtually all Italian citizens aged 0–35 years have HBV vaccination coverage [21], whereas none of the 1,212 undocumented immigrants or refugees in our study received active immune-prophylaxis against HBV nor had they been tested for HBV markers after an average stay in Italy of 4.5 years. Taking care of this vulnerable group of individuals should be a moral duty for every government or national healthcare system in developed countries [22, 47]. Extending monitoring and treatment of HBV chronic infection and HBV universal vaccination to undocumented immigrants and low-income refugees is a mandatory epidemiological approach towards eradicating HBV infection in this vulnerable group and in their host countries.

## Additional file

Additional file 1: Multilingual abstracts in the five official working languages of the United Nations. (PDF 626 kb)

## Abbreviations

AHB: Acute hepatitis B
ALT: Alanine aminotransferase
CI: Confidence interval
HBc: Hepatitis B core antibody
HBe: Hepatitis Be antibody
HBeAg: Hepatitis B envelope antigen
HBs: Hepatitis B surface antibody
HBsAg: Hepatitis B surface antigen
HBV: Hepatitis B virus
HCC: Hepatocellular carcinoma
HCV: Hepatitis C virus
HIV: Human immunodeficiency virus
OR: Odds ratio
SD: Standard deviation
SSA: Sub-Saharan Africa
