# Supplementary material for: Hepatitis B virus infection in undocumented immigrants and refugees in Southern Italy: demographic, virological, and clinical features
Source: Infect Dis Poverty. 2017 Feb 9;6:33. doi: 10.1186/s40249-016-0228-4 (PMC5299765; doi:10.1186/s40249-016-0228-4)
Supplement: Additional file 1: — Multilingual abstracts in the five official working languages of the United Nations. (PDF 626 kb) [file 40249_2016_228_MOESM1_ESM.pdf]

Translation of the abstract into the five official working languages of the United Nations

الديموغرافية والفيروسية الخصائص :عدوى فيرس التهاب الكبد بي بين المهاجرين غير الشرعيين واللاجئين في جنوب إيطاليا والسريرية

نيكولا كوبولا ولوريدانا آليسيو ولوسيانو جوالديري وماريانونييتا بيساتيرو وكاترينا ساجنيللي وكارمين مينتشيني وجيوفاني دي لو وجيوسيبيكابريو وماريو ستاراس ولورينزو أونوراتو وجيوسيبي سيجنوريللو ومارغريتا ماسيرا وإيتالو فرانسيسكو أنجيليلو باسكال وإيفانجيليستا ساجنيللي

موجز

نبذة عامة: تعد البيانات الواردة بشأن عدوى فيرس التهاب الكبد بي ضئيلة، وإنما كان الهدف من هذه الدراسة هو تحديد الخصائص الديموغرافية والفيروسية والسريرية للأشخاص المصابين بعدوى مزمنة من التهاب الكبد بي في مجموعة من المهاجرين المقيمين في نابولي، إيطاليا.

الوسائل: تم تقديم عرض بالكشف عن عدوى فيرس التهاب الكبد بي لـ 1331 من المهاجرين، وافق منهم 1212) بنسبة 91 (% 831مهاجرًا غير شرعي و 381 لاجئًا) وتم الكشف عنهم باختبار المستضد السطحي لالتهاب الكبد بي والأجسام الأساسية المضادة لالتهاب الكبد بي، وبعد ذلك تم إجراء فحص إضافي لأولئك الذين كانت نتائجهم إيجابية في الوحدات الخاصة بالأمراض المعدية من المستوى الثالث.

النتائج: تبين أنه من بين الـ 1212 مهاجرا كان هناك 116 بنسبة) فردا نتائجهم إيجابية 9.6 (% في اختبار المستضد السطحي لالتهاب الكبد بي بينما كانت نتائج 490 فردا سلبية في اختبار المستضد السطحي وإيجابية في اختبار الأجسام الأساسية المضادة لالتهاب الكبد بي 40.4 بنسبة) (% وأخيرا كانت نتائج 606 بنسبة) فردا سلبية المصل في كلا الاختبارين 50 (%. علاوة على ذلك، كانت نتائج 21 بنسبة) فردا إيجابية في اختبار مضاد فيروس نقص المناعة البشرية 1.7 (% ونتائج 45 فردا إيجابية في اختبار الأجسام المضادَّة لفيروس التهاب الكبد سي بنسبة 3.7 (%. وأوضح تحليل الانحدار أن الذكور (أو: 1.79 ;95% *CI*: 1.28–2.51) والمنشأ الإفريقي في جنوب الصحراء الكبرى (أو: 6.18 ;95% *CI*: 3.37–11.36) وتدني المستوى التعليمي (أو: 0.96 ;95% *CI*: 0.94–0.99) ومخاطر الحقن الطفيفة التي تؤدي للإصابة بعدوى فيروس التهاب الكبد بي (الوخز بالإبر، والوشم، والثقب، والممارسات القبلية، أو: 1.54 ;95% *CI*: 1،1 حتي 2،16) ارتبطت بشكل مستقل بالإصابة بعدوى فيروس التهاب الكبد بي المستمر أو الماضي. من بين 116 فردا من المهاجرين المصابين بالتهاب الكبد بي، أجرى 90 بنسبة) 77.6 (% منهم مسارهم التشخيصي في وحدة خاصة بالأمراض المعدية من المستوى الثالث: و 29 (32.2 (% كانت لديهم أعراض فيروس التهاب الكبد بي، ونواقل غير متناظرة لفيروس 43 (47.8 (% كانت لديهم أعراض نواقل متناظرة لفيروس الكبد بي، و 14 (15.6 (% كان لديهم التهاب كبد مزمن، و 4 (4.4 (% كان لديهم تليف الكبد، كما وجد حالتان مصاباتان بسرطان كبد متراكب.

الاستنتاجات: توضح البيانات الخصائص الديموغرافية والسريرية والفيروسية لعدوى فيروس التهاب الكبد بي في المهاجرين في إيطاليا، وتظهر حاجة السلطات الصحية الايطالية إلى تعزيز دعمهم لتوفير الفحص والتطعيم ضد الالتهاب الكبدي الوبائي، وعلاجه، وتوفير برامج تعليمية لأولئك الأشخاص.

Translated from English version into Arabic by Mohamed Mortada, through

TRANSLATORS
WITHOUT BORDERS

意大利南部的无证移民和难民中乙肝病毒的感染情况：人口学、病毒学和临床特征

Nicola Coppola, Loredana Alessio, Luciano Gualdieri, Mariantonietta Pisaturo, Caterina Sagnelli, Carmine Minichini, Giovanni Di Caprio, Mario Starace, Lorenzo Onorato, Giuseppe Signoriello, Margherita Macera, Italo Francesco Angelillo, Giuseppe Pasquale, Evangelista Sagnelli

摘要

引言：目前，移民人群的乙肝病毒（HBV）的感染研究数据很少。本研究旨在对意大利那不勒斯移民人群进行队列研究，分析该人群的乙肝病毒感染的人口学，病毒学和临床的特征。

方法：对 1 331 名移民进行 HBV 感染筛查，其中，1 212 人（91%）（831 名无证移民和 381 名难民）接受了乙肝表面抗原(HBsAg)和乙肝核心抗体(HBc)的筛查。HbsAg 阳性者在第三级传染病单位接受诊断。

结果：在筛查的 1 212 名移民中，116 例（9.6%)为 HbsAg 阳性，490 例(40.4%)为 HbsAg 阴性/抗-HBc 阳性，606 人(50%)上述两个血清学反应均为阴性。此外，21 例(1.7%)抗 HIV 阳性，45 人(3.7%)抗丙肝病毒阳性。Logistic 回归分析显示，男性(*OR*: 1.79; 95%*CI*: 1.28–2.51)、撒哈拉以南非洲裔(*OR*: 6.18; 95%*CI*: 3.37–11.36)、低学历(*OR*: 0.96; 95%*CI*: 0.94–0.99)，以及轻度非胃肠的因素（针灸、纹身、穿孔以及族群活动, *OR*: 1.54; 95%*CI*: 1.1–2.16）与 HBV 既往感染和持续感染有关。116 例 HbsAg 阳性移民中，90 例(77.6%)在第三级传染病单位完成诊断：其中 29 例(32.2%)是无症状的非病毒血症 HbsAg 携带者，43 例 (47.8%)为无症状的病毒血症携带者，14 例(15.6%)为慢性肝炎患者，4 例(4.4%)患有肝硬化， 2 例叠加患肝细胞癌。

结论：本研究数据阐明了意大利移民人群中的乙肝感染的人口学、临床和病毒学特征，同时，研究表明，意大利卫生行政部门需加强对移民人群的筛查、HBV 疫苗接种、治疗和健康教育。

Translated from English version into Chinese by Hong Tu, edited by Pin Yang

Infection par le virus de l'hépatite B chez les réfugiés et les immigrés sans papier dans le Sud de l'Italie: caractéristiques démographiques, cliniques et virologiques

Nicola Coppola, Loredana Alessio, Luciano Gualdieri, Mariantonietta Pisaturo, Caterina Sagnelli, Carmine Minichini, Giovanni Di Caprio, Mario Starace, Lorenzo Onorato, Giuseppe Signoriello, Margherita Macera, Italo Francesco Angelillo, Giuseppe Pasquale, Evangelista Sagnelli

RÉSUMÉ

Contexte: Les données sur l'infection par le virus de l'hépatite B (VHB) chez les immigrés sont sommaires. L'objectif de cette étude est de définir les caractéristiques démographiques, cliniques et virologiques des sujets frappés d'infection chronique par le VHB dans un groupe d'immigrés vivant à Naples, en Italie.

Méthodes: Un test de dépistage de l'infection par le virus de l'hépatite B a été proposé à 1 331 immigrés, 1 212 (91%) (831 immigrés sans papier et 381 réfugiés) ont accepté et on a analysé l'antigène de surface de l'hépatite B (HbsAg) et l' a*nticorps dirigé contre* l'*antigène* de surface

du *virus* de l'*hépatite B (Hbc). Les personnes positives à l'HbsAg ont été examinées dans des unités de maladies infectieuses de troisième degré.*
Résultats: Parmi les 1 212 immigrés analysés, 116 (9,6%) étaient *HbsAg positifs, 490 (40,4%) étaient HbsAg négatifs/anti-HBc positifs et 606 (50%) étaient séronégatifs pour les deux tests. De plus, 21 personnes (1,7%) étaient anti-VIH positives et 45 (3,7%) étaient anti VHC positives. L'analyse de régression logistique a montré que* le sexe masculin (*OR*: 1.79; 95%*CI*: 1.28–2.51), l'origine d'Afrique subsaharienne (*OR*: 6.18; 95%*CI*: 3.37–11.36), le faible niveau d'instruction (*OR*: 0.96; 95%*CI*: 0.94–0.99) et d'autres risques parentéraux mineurs de contracter l'infection par le VHB (acupuncture, tatouage, piercing, ou pratiques tribales, *OR*: 1.54; 95%*CI*: 1.1–2.16) étaient indépendamment associés à une infection par le VHB présente ou passée. Chez lez 116 immigrés *HbsAg positifs, 90 (77,6%) ont complété leur itinéraire diagnostique dans une unité de maladies infectieuses de troisième degré*: 29 (32.2%) étaient des porteurs *HbsAg* asymptomatiques non virémiques, 43 (47.8%) étaient des porteurs asymptomatiques virémiques, 14 (15.6%) avaient une hépatite chronique et 4 (4.4%) souffraient d'une cirrhose du foie, dont 2 avec carcinome hépatocellulaire superposé.
Conclusions: Ces données illustrent les caractéristiques démographiques, cliniques et virologiques de l'infection par le VHB chez les immigrés en Italie et indiquent la nécessité, pour les autorités italiennes, de renforcer l'aide destinée à fournir le dépistage, la vaccination, le traitement contre le VHB et des programmes de formation pour ces populations.

Translated from English version into French by Julie Thonus, through

TRANSLATORS
WITHOUT BORDERS

Вирусная инфекция гепатита В среди нелегальных иммигрантов и беженцев на юге Италии: демографические, вирусологические и клинические характеристики

Никола Коппола, Лоредана Алессио, Люсиано Гуалдьери, Мариантуанетта Писатуро, Катерина Саньелли, Кармэн Миничини, Джованни Ди Каприо, Марио Старасе, Лоренцо Онорато, Джузеппе Синьорелло, Маргарита Масера, Итало Франческо Анджелилло, Джузеппе Паскале, Еванджелиста Саньелли

КРАТКОЕ ИЗЛОЖЕНИЕ

История вопроса: Данные о вирусной инфекции гепатита В (HBV) среди иммигрантов весьма ограниченны. Целью данного исследования стало определение демографических, вирусологических и клинических характеристик пациентов, зараженных хроническим HBV, среди иммигрантов, проживающих в Неаполе (Италия).
Методы: Обследование на инфекцию гепатита В было предложено 1331 иммигранту, из которых 1212 (91%) (831 нелегальных иммигрантов и 381 беженец) согласились на него. Были проведены анализы на поверхностный антиген гепатита В (HBsAg) и антитело к

HBcAg (HBc). Пациенты с положительным результатом на HBsAg далее наблюдались в инфекционных учреждениях третьего уровня.
Результаты: Из 1212 прошедших обследование иммигрантов, 116 (9,6%) оказались HBsAg-положительными, 490 (40,4%) – HBsAg-отрицательными и HBc-положительными, а 606 (50%) – сероотрицательными по обоим факторам. Кроме этого, 21 человек (1,7%) имел положительный результат по HIV, а 45 (3,7%) – по гепатиту С. Логистический регрессивный анализ показал, что принадлежность к мужскому полу (*OR*: 1,79; 95% *CI*: 1,28–2,51), африканское происхождение к югу от Сахары (*OR*: 6,18; 95% *CI*: 3,37–11,36), низкий уровень образования (*OR*: 0,96; 95% *CI*: 0,94–0,99), незначительные факторы родительского риска заражения HBV (иглоукалывание, татуировки, пирсинг или племенные традиции, *OR*: 1,54; 95% *CI*: 1,1–2,16) были независимо связаны с текущим или прошлым заражением HBV. Из 116 иммигрантов с положительной реакцией на HBsAg, 90 (77,6%) завершили прохождение диагностики в инфекционных учреждениях третьего уровня: 29 (32,2%) были бессимптомными незараженными носителями HBsAg, 43 (47,8%) – бессимптомными зараженными носителями, у 14 (15,6%) был хронический гепатит, а у четырех (4,4%) – цирроз печени с гепатоцеллюлярной карциномой второй стадии.
Заключение: Эти данные иллюстрируют демографические, клинические и вирусологические характеристики инфицирования HBv среди иммигрантов в Италии и указывают на необходимость расширения органами здравоохранения Италии поддержки обследований, вакцинации против HBV, лечения и предоставления образовательных программ для этой группы населения.

Translated from English version into Russian by Elena McDonnell, through

TRANSLATORS
WITHOUT BORDERS

Infección por virus de la hepatitis B entre inmigrantes indocumentados y refugiados en el sur de Italia: aspectos demográficos, virológicos y clínicos

Nicola Coppola, Loredana Alessio, Luciano Gualdieri, Mariantonietta Pisaturo, Caterina Sagnelli, Carmine Minichini, Giovanni Di Caprio, Mario Starace, Lorenzo Onorato, Giuseppe Signoriello, Margherita Macera, Italo Francesco Angelillo, Giuseppe Pasquale, Evangelista Sagnelli

RESUMEN

Antecedentes: Los datos sobre la infección por el virus de la hepatitis B (VHB) entre la población inmigrante son escasos. El propósito de este estudio es definir los aspectos demográficos, virológicos y clínicos de los sujetos con infección crónica por VHB entre una cohorte de inmigrantes residentes en Nápoles, Italia.
Métodos: Se ofreció una prueba de cribado a 1 331 inmigrantes, de los cuales 1 212 (el 91 %: 831 inmigrantes indocumentados y 381 refugiados) aceptaron y fueron examinados para detectar la presencia del antígeno de superficie del VHB (HBsAg) y del anticuerpo central contra la hepatitis

B (HBc). Aquellos con positividad para HBsAg fueron objeto de un examen más detallado en servicios de enfermedades infecciosas de nivel 3.

Resultados: De los 1 212 inmigrantes sometidos al estudio de cribado, 116 (9,6 %) revelaron seropositividad para HBsAG, 490 (40,4 %) negatividad para HBsAG y positividad para anti-HBc y 606 (50%) negatividad para ambos. Además, 21 (1,7%) eran seropositivos para el virus de la inmunodeficiencia humana y 45 (3,7 %) para el de la hepatitis C. El análisis de regresión logística reveló que el sexo masculino (*OR*: 1,79; 95%*CI*: 1,28-2,51), el origen africano subsahariano (*OR*: 6,18; 95%*CI*: 3,37-11,36), el nivel educativo bajo (*OR: 0,96;* 95%*CI*: 0,94-0,99) y el riesgo leve de infección parenteral por VHB (acupuntura, tatuajes, *piercings* o prácticas tribales, *OR*: 1,54; 95%*CI*: 1,1-2,16) se encontraban asociados independientemente con la infección por VHB actual o pasada. De los 116 inmigrantes con presencia de HBsAg en sangre, 90 (77,6 %) completaron su itinerario diagnóstico en servicios de enfermedades infecciosas de nivel 3: 29 (32,2 %) eran portadores asintomáticos no virémicos de HBsAg, 43 (47,8 %) eran portadores asintomáticos virémicos, 14 (15,6 %) padecían hepatitis crónica, y 4 (4,4 %) cirrosis hepática, dos de los cuales también presentaban carcinoma hepatocelular.

Conclusiones: Los datos ilustran las características demográficas, clínicas y virológicas de la infección por VHB entre los inmigrantes de Italia y resaltan la necesidad de que las autoridades sanitarias italianas refuercen su apoyo para proporcionar más pruebas de cribado, vacunas contra el VHB, tratamientos y programas educativos para estos grupos de población.

Translated from English version into Spanish by Gloria Estevan Alcaide, through

TRANSLATORS
WITHOUT BORDERS
